# Allelic Variation of *Glu-A1* and *Glu-B1* Genes in Winter Durum Wheat and Its Effect on Quality Parameters

**DOI:** 10.3390/foods12071436

**Published:** 2023-03-28

**Authors:** Aleksandra Yu. Kroupina, Aleksey S. Yanovsky, Varvara A. Korobkova, Ludmila A. Bespalova, Andrey V. Arkhipov, Galina I. Bukreeva, Anastasiya D. Voropaeva, Pavel Yu. Kroupin, Dmitry Y. Litvinov, Aleksandra A. Mudrova, Daniil S. Ulyanov, Gennady I. Karlov, Mikhail G. Divashuk

**Affiliations:** 1 All-Russia Research Institute of Agricultural Biotechnology, 127550 Moscow, Russia; 2 P.P. Lukyanenko National Grain Centre, Central Estate of KNIISH, 350012 Krasnodar, Russia

**Keywords:** durum wheat, pasta quality, gluten index, gluten content, storage protein subunits, high-molecular-weight glutenins, KASP markers, SDS-PAGE analysis

## Abstract

Winter durum wheat is a relatively young crop that is highly adaptable due to its winter type of growth habit. The priority of breeding and genetic improvement of winter durum wheat is to improve grain quality and pasta quality, largely determined by the glutenin storage proteins. In the present study, a collection of 76 accessions of winter durum wheat from P.P. Lukyanenko National Grain Centre was studied. The allelic state of high-molecular-weight glutenin genes, *Glu-A1* and *Glu-B1*, using PCR markers and SDS-PAGE was identified and grain and pasta quality traits were assessed in a two-year field experiment. The positive effect of the *Glu-A1a* allele and a negative effect of *Glu-A1c* on the gluten index were shown. It was found that *Glu-B1al* and *Glu-B1f* have a positive effect on the quality and quantity of protein and gluten, while the *Glu-A1c* + *Glu-B1al* genotypes were closest to the high-quality category in protein-associated quality traits.

## 1. Introduction

Durum wheat (*Triticum turgidum* L. ssp. *durum* Desf. Husn) is used in the food industry to make pasta, bulgur and couscous. Pasta, owing to its long shelf life, low price and ease of preparation, is characterised by a high level of consumption worldwide, including in Russia. In 2021, about 735 thousand tons of durum wheat grain were harvested in Russia and the sown area in 2022 was projected at 790 thousand ha [1]. Pasta sales in Russia grew at an annual rate of 2.1–3.2% in 2016–2020; in 2020, pasta sales reached 1442 thousand tons, the highest level in a decade [2]. In 2021, Russia ranked 15th in the world in pasta consumption and 6th in production [3].

The quality of wheat products is an integral indicator that is determined by the properties of starch, protein, puroindolines, pigments and their interactions [4,5]. Durum wheat has a number of technological properties that make it ideal for the manufacturing of pasta products. The importance of the quality of raw materials for these products is constantly increasing due to the increasing requirements from producers and consumers [6]. The main parameters affecting the quality of final durum wheat products are test weight (TW), kernel vitreousness (KV), yellow pigment content (YPC) and grain protein content (GPC) [7,8]. The yellow pigments not only make pasta products more attractive for consumers but also have a positive effect on human health [9,10]. The high protein content creates a strong gluten matrix that retains the starch molecules during cooking and prevents the pasta surface from becoming sticky and retains its shape [11,12].

Pasta quality is determined not only by the protein content but also by the quality of the high- and low-molecular-weight glutenins (LMW and HMW) encoded by the *Glu-1* and *Glu-3* loci, respectively. Although LMW glutenins have a dominant effect over the HMW-GS in durum wheat, the HMW-GS allelic state has, on average, a considerable effect on gluten quality. The *Glu-A1* allelic state has no significant effect on gluten quality, but several studies have shown that allele *Glu-A1c* (null subunit) has a negative effect, while allele *Glu-A1b* (protein subunit 2∗) is associated with a higher gluten strength than allele *Glu-A1b* (subunit 1). Among the *Glu-B1* allelic variants, alleles *Glu-B1b* (subunits Bx7 + By8), *Glu-B1d* (subunits Bx6 + By8, (Bx6 having a stronger effect on gluten strength than By8 [13])), *Glu-B1bc* (subunits Bx6 + By17) and h (subunits Bx14 + By15) in contrast to allele *Glu-B1a* (subunit Bx7). However, alleles such as *Glu-B1f* (subunits Bx13 + By16) and *Glu-B1e* (subunits Bx20 + By20) in different studies have multidirectional effects on gluten, which may be due to genetic background and cultivation conditions (see review [14]). The described correlations are relevant for spring durum wheat and may have other directions in winter durum wheat germplasm.

In Russia, a decrease in the area under spring durum wheat due to its lower yields has led to the production of pasta from common wheat, which has had a negative impact on its quality [15]. Winter durum wheat in Central and Eastern Europe is sown in autumn and gains access to more moisture accumulated in the soil due to autumn and winter precipitation [16]; it undergoes the stages of grain setting to grain filling under less stressed hydrothermal conditions, thus the yield potential is higher than that of spring wheat [17,18]. To improve the competitiveness of winter durum wheat relative to spring wheat, breeding centres in southern Russia are actively performing breeding for quality [19,20,21,22]. Commercially successful wheat cultivars have been developed by the P.P. Lukyanenko National Grain Centre (NGC) in Krasnodar for many decades [23,24,25,26,27]. The collection of winter durum wheat cultivars and lines developed at NGC is of particular interest for study as an example of highly adaptive local genotypes selected for specific agroclimatic conditions [28,29,30]. In addition, winter durum wheat is a relatively young and insufficiently studied crop that forms grain under conditions other than spring durum wheat; hence, the effect of HMW-GS alleles on pasta quality may be different. Thus, studying the allelic structure of HMW-GS genes and the effect of the allelic state of *Glu-1* on the quality of durum wheat in local collections is an important and timely issue, especially for germplasm representing regional breeding programmes where rare alleles or their atypical effects on quality may be identified [31,32].

## 2. Materials and Methods

### 2.1. Plant Material

The list of winter durum wheat accessions representing commercial cultivars and candidate cultivars for registration from the NGC’s competitive variety trial nursery in the present study is shown in Table 1. The origin and year of release are shown in Supplementary Table S1.

### 2.2. Field Experiment

The field experiment was performed in a plot of land at the P.P. Lukyanenko National Grain Centre (45°03′40″ N 38°54′23″ E). Under the main tillage, the complex fertilizer Azofoska was applied (N16:P16:K16, 200 kg/ha). Ploughing was performed to a depth of 20–25 cm with a turn-wrest plough (Lemken GmbH & Co, Alpen, Germany), followed by double discing with a Rubin disc harrow (Lemken GmbH & Co, Alpen, Germany). Sowing was performed using a self-propelled Plotseed TC seed drill (Wintersteiger, Ried, Austria) in 5 m^2^ plots in double replication. Two top dressings were carried out using nitrate (N 34, 150 kg/ha): the first after the resumption of spring vegetation (150 kg per ha), the second in the booting stage. Harvesting was performed by direct combining using a Classic harvesting machine (Wintersteiger, Ried, Austria).

### 2.3. Grain Quality Evaluation

A 500 g sample of the whole grain was used to measure protein content, gluten content and SDS sedimentation volume using an Infratec™ 1241 grain analyser (Foss Analytical, Hillerød, Denmark) according to the manufacturer’s recommendations. The principle of the device is based on using near-infrared light transmitted through the grain [33].

A Perten Glutomatic^®^ 2100 System was used, according to the manufacturer’s instructions, to determine the gluten index. Briefly, 10 g ± 0.01 g of whole meal is placed into the Glutomatic wash chamber with an 88-micron polyester sieve and a 4.8 mL 2% sodium chloride solution is added and mixed for 20 s to form a dough. After termination of the mixing phase, the washing automatically starts and continues for five minutes. The undivided wet gluten piece is transferred to the special sieve cassette and 30 s after completed washing it is centrifuged for one minute at 6000 ± 5 rpm in a Centrifuge 2015. The fraction that passes through the sieves is scraped off with a spatula and weighed. The fraction remaining on the inside of the sieve is collected and added to the balance. The total wet gluten weight is obtained. The amount of gluten remaining on the centrifuge sieve in relation to the total wet gluten weight is the gluten index.

### 2.4. Pasta Quality Evaluation

The pasta was made from durum wheat grain, as described in [34]. A sample of pasta (25 g) is dipped into a 500 mL measuring cylinder filled with 300 mL room temperature water. The cylinder is then shaken to remove air bubbles. The volume of the products taken is determined by the rising water level. After measuring the volume of the drained water, the pasta is transferred into a 1000 mL conical flask and boiling distilled water (300 mL) is poured in the bath (water temperature 97–98 °C), where they cook for 20 min. At the end of cooking, the pasta is transferred to a sieve and after the excess water has drained off, the volume of the cooked products is determined using the above-described method. The volume increase index is calculated as the relation of the volume of dry pasta to the volume of pasta after cooking.

Pasta breaking strength was determined using apparatus IPM-1 (VNIIZ, Moscow, Russia). A pasta tube is placed in the notches of the stand. With continuous movement of the chain from top to bottom, the breaker bar is lowered gently onto the pasta tube. The load is evenly increased until the pasta tube breaks. The load is determined by reading the arrow on the dial at the moment the tube breaks. Ten pasta tubes are taken from each sample and their strength is examined. The average of the 10 readings describes the strength of the pasta. The following scale was used for the breaking strength estimation: “5” (excellent), 800 g and more; “4” (good), 750–799 g; “3.5” (quite satisfactory), 700–749 g; “3” (satisfactory), 600–699 g; “2” (unsatisfactory), less than 600 g (Table 1).

The general pasta estimation was graded as an integral score summarizing the following parameters: breaking strength, volume increase index and pasta colour: “5” (excellent), breaking strength 800 g and more, volume increase index 3.5–4.0, lime–yellow or yellow pasta colour; “4” (good), breaking strength 750–799 g, volume increase index 4.1–4.5, cream pasta colour; “3” (medium), breaking strength 700–749 g, volume increase index 4.6–5.0, yellow and yellow–cream pasta colour; “2” (bad), breaking strength less than 700 g, volume increase index 5.1 and more, white and grey pasta colour.

### 2.5. High Molecular Glutenin Characterisation

The protein fractions from flour samples were extracted using a modified procedure described in [35]. The endosperm of a single wheat kernel is crushed. First, for a 96-well plate, gliadins are extracted in a stirred BioSan water bath WB-4MS (BioSan, Riga, Latvia) at 65 °C for 30 min in 1.0 mL of A-solution (50% propanol-2 (*v/v*)) with intermittent shaking (twice) followed by centrifugation in a 5417R centrifuge (Eppendorf, Hamburg, Germany) for 4 min at 2000 rpm at room temperature; the supernatant is discarded. After repeating this extraction once more, the residue is washed in 0.5 mL of A-solution, centrifuged for 10 min at 2000 rpm at room temperature and then all the liquid is removed. Then, glutenin is extracted within 30 min at 65 °C from the residue in 0.1 mL of B-solution (50% propanol-2 (*v*/*v*), 1 M Tris-HCl, pH 8.0) containing 1% (*w*/*v*) freshly added dithiothreitol. After a 10 min centrifugation at 2000 rpm at room temperature, 0.1 mL of B-solution containing 1.4% (*v*/*v*) freshly mixed 4-vinylpyridine is added to each tube and incubated for 15 min at 65 °C for protein alkylation. The sample is then centrifuged for 7 min at 2000 rpm at room temperature and 0.1 mL of supernatant is transferred to a new tube containing 0.1 mL of C-solution (2% SDS (*w*/*v*), 40% glycerol (*w*/*v*), bromophenol blue, 1 M Tris-HCl, pH 8.0), shaken briefly and then incubated within 15 min at 65 °C for the complexing of SDS with the reduced and alkylated glutenin polypeptides. After 2 min of centrifuging at 2000 rpm at room temperature, 14–17 µL of the supernatant are loaded into a sample well of the gel for the SDS-PAGE separation of the glutenin subunits. Electrophoresis is performed using an omniPAGE mini wide vertical protein electrophoresis system (Cleaver Scientific Ltd., Rugby, UK) with plate dimensions 20 × 10 cm. The lower separating gel has a concentration of acrylamide of 8% and is prepared using 5625 µL of 1.5 M Tris-HCl (pH 8.8), 6000 µL of 30% acrylamide solution, 900 µL of 10% SDS, 9825 µL of dH_2_O, 150 µL of 10% APS solution and 15 µL of TEMED on two 0.75 mm thickness gels. The upper stacking gel has a concentration of acrylamide of 4% and is prepared using 2250 µL of 0.5 M Tris-HCl (pH 6.8), 1200 µL of 30% acrylamide solution, 360 µL of 10% SDS solution, 5091 µL of dH_2_O, 90 µL of 10% APS solution and 9 µL of TEMED on two 0.75 mm thickness gels. Gels are run at 60 V before the sample enters the separating gel and then at 120 V approximately for 2–3 h. The gels are stained using Coomassie blue. Gels are documented at ChemiDoc Imaging System (Bio-Rad Laboratories Inc., Hercules, CA, USA).

### 2.6. Glu-A1 and Glu-B1 Genotyping

The genomic DNA was extracted from the dried leaves of four-day-old seedlings, as described in [36]. For the detection of the allelic state of *Glu-1,* loci KASP markers developed in [37] are used. For the *Glu-A1* allelic state, the following primers are used: FAM primer AAGTGTAACTTCTCCGCAACG for *Glu-A1c* (null allele); HEX primer ACCTAAGTGTAACTTCTCCGCAACA for *Glu-A1a*/*Glu-A1b* (subunits Ax1/Ax2*); common primer CGAAGAAGCTTGGCCTGGATAGTAT. For the *Glu-B1* allelic state, the following primers were used: FAM primer GTGGAATATTAGTGATGGCGTGAG for non-*Glu-B1al* (subunits other than Bx7^OE^); HEX primer GTGGAATATTAGTGATGGCGTGAC for *Glu-B1al* (subunit Bx7^OE^). PCR is performed in a 96-well PCR plate in a total volume of 10 μL containing 5 μL of master mix (LCG Biosearch Techhnologies, Berlin, Germany; KASP 2X Mmix KBS-1050-102 including the fluorescent dyes FAM, HEX, ROX), 1.5 pM μL of allele-specific primers, 3.75 pM of common primer and 5 μL of DNA template (50 ng per well). For PCR, a touchdown protocol is used starting with a 15 min hot enzyme activation at 94 °C, followed by 10 cycles of 94 °C for 20 s, 61–55 °C for 60 s (0.6 °C/cycle) and 94 °C for 30 s. This is continued for 35 cycles of 94 °C for 20 s and 55 °C for 60 s, followed by a plate reading at 37 °C for 60 s. PCR is performed in BIO-RAD CFX96 (Bio-Rad Laboratories Inc., Hercules, CA, USA) using Bio-Rad CFX Manager 3.1 software. Based on the comparison of the results of the KASP and SDS-PAGE analyses, the allelic state of *Glu-A1* and *Glu-B1* is concluded.

### 2.7. Statistical Analyses

For all observed values expressed in percent (gluten content, protein content, gluten index), logit transformation was applied, then all statistical calculations were performed with the transformed values. By transforming percentages into logarithms of odds, the logit transformation enhances the statistical properties of a variable and stabilizes variances, which increases the accuracy of coefficient estimates. The resulting transformed data become more suitable for regression models with better statistical properties. Furthermore, the logit transformation can make the relationships between variables linear and symmetrical, which can simplify analyses even further [38]. The scores of general pasta estimation, based on a five-point scale, were grouped based on the kNN method, with the number of groups being selected by its silhouette metric. The resulting groups were further named according to the corresponding intervals of scores and treated as ordinal categorical data. For the various states of the input categories, the significance of the differences of mean of the observed quantitative values was estimated according to Tukey’s paired criterion at a 95% confidence interval. The results of the calculation of the Tukey’s criterion, as well as the calculated expected differences of the means, were supplemented with calculations of the mean for each subcategory and the general mean for both categories; the expected differences between the means were also presented as a percentage from the general mean for both categories. The mean observed values for each subcategory are given in the table, with the indexes of the corresponding groups according to Tukey’s criterion (Table 2).

Cross- and internal correlations of the observed values and the input categories, ordered and unordered, were estimated using the Φk coefficient [39]. Estimations of the correlation coefficients and their significances are presented in the diagrams in Figure 3.

Before the principal component analysis (PCA), the numerical columns missing over 30% of entries were excluded from the analysis; otherwise, they were filled with respective median values for the whole set of observations. The remaining values were normalized from 0 to 1. For PCA, based on the obtained array, three first components were calculated in three iterations. On the basis of pairs of received components, diagrams were plotted, containing the projections of the vectors of the normalized observed value correlations, as well as the data points with the indication of the corresponding categories and the ellipse outlines of the areas of their greatest density; the percentages of variations explained by components are given in the diagrams (Figure 4).

## 3. Results

### 3.1. Structure of the Collection

**Figure 1:**
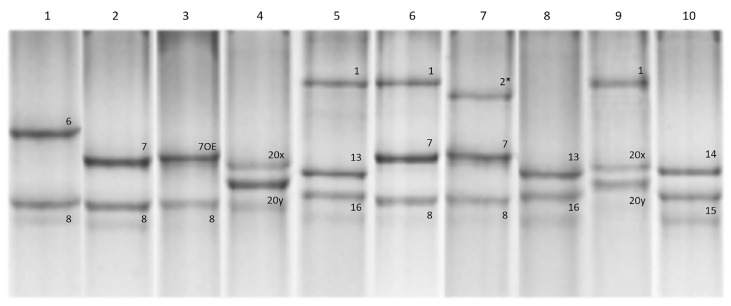
Patterns of HMW-GS subunit compositions in the studied collection of winter durum wheat as revealed using SDS-PAGE analysis. Accessions are as follows: 1, 3680h62; 2, 3591h96; 3, MV Hundur; 4, MVTD 15-98; 5, Yakhont (760/10); 6, Yubilarka (330/10); 7, 3561h28; 8, 3796h33; 9, Donchanka; 10, MV Pennedur; 2* denotes subunit Ax2*.

**Figure 2:**
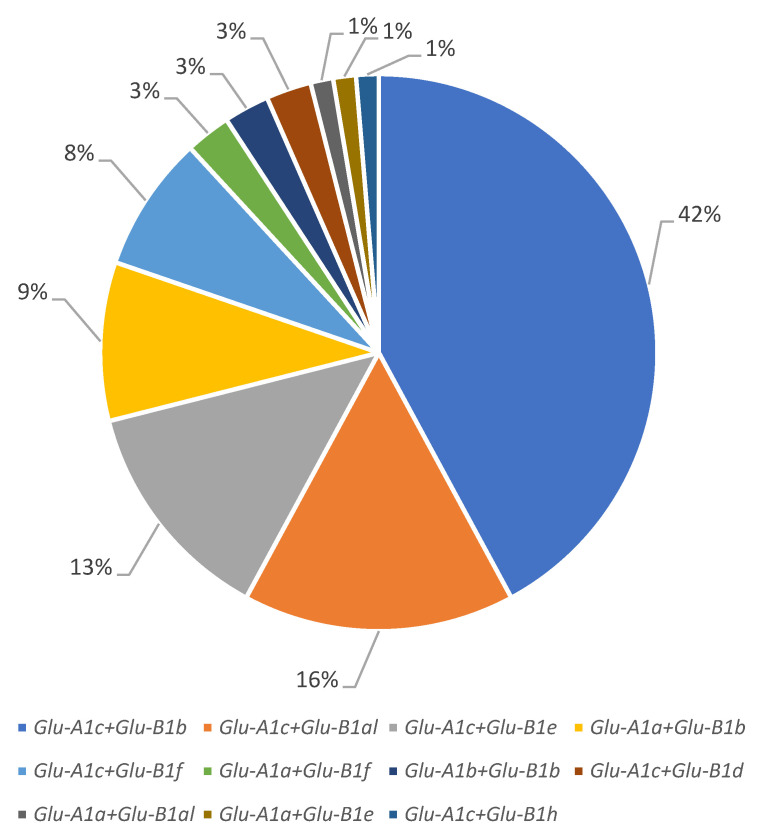
Structure of the winter durum wheat collection by allelic variants of the *Glu-A1* and *Glu-B1* loci.

Most of the accessions from the studied collection (63 accessions) carried the *Glu-A1c* allele, followed by *Glu-A1a* (11 accessions) and *Glu-A1b* (2 accessions). At the *Glu-B1* locus, the *Glu-B1b* allele was predominant (41 accessions), *Glu-B1al* and *Glu-B1e* were almost equally represented (13 and 11 accessions, respectively) and *Glu-B1f*, *Glu-B1d* and *Glu-B1h* were found in 8, 2 and 1 accessions, respectively (Figure 1 and Figure 2; Table 1).

The allelic combination *Glu-A1c* + *Glu-B1b* had the highest proportion (32 accessions), combinations *Glu-A1c* + *Glu-B1al* and *Glu-A1c* + *Glu-B1e* were approximately equally represented (12 and 10 accessions, respectively) and *Glu-A1a* + *Glu-B1b* and *Glu-A1c* + *Glu-B1f* also had approximately equal lower frequencies (7 and 6 accessions, respectively); each of *Glu-A1a* + *Glu-B1f*, *Glu-A1b* + *Glu-B1b* and *Glu-A1c* + *Glu-B1d* were found in two cultivars. Each of the *Glu-A1a* + *Glu-B1al*, *Glu-A1a* + *Glu-B1e* and *Glu-A1c* + *Glu-B1h* genotypes were identified in only one cultivar and therefore excluded from the analysis.

### 3.2. Associations between Quality Parameters

According to the general pasta estimation, all accessions were divided into three quality categories: high (4.3–4.5, HQ), medium (3.8–4.1, MQ) and low (3.0–3.5, LQ). The quality category showed a strong negative correlation with the volume increase index of pasta in both years (Φk 0.76 in 2019–2020 and 0.83 in 2020–2021, Figure 3; opposite direction in the PCA diagram, Figure 4) and accessions classified as HQ in both years had a statistically significant lower volume increase index (3.60 and 3.70 units) compared with MQ (lower by 0.33 and 0.32 units, or 9.2% and 8.6%) and LQ accessions (0.52 and 0.60 units lower, or 14.4% and 16.2%, Table 2). In 2019–2020, a positive relationship was shown between the quality category on one side and the protein and gluten content on the other side (same direction in the PCA diagram, Figure 4). HQ accessions had a significantly higher gluten content compared with MQ and LQ accessions by 1.2 pp (5.0%) and 1.7 pp (7.0%) and a higher protein content compared with LQ accessions by 0.6 pp (4.3%, Table 2). The highest volume of gluten index observed in MQ accessions was higher than in HQ and LQ accessions by 15.0 and 21.4 units (30.6% and 33.4%), respectively (Table 2).

**Figure 3:**
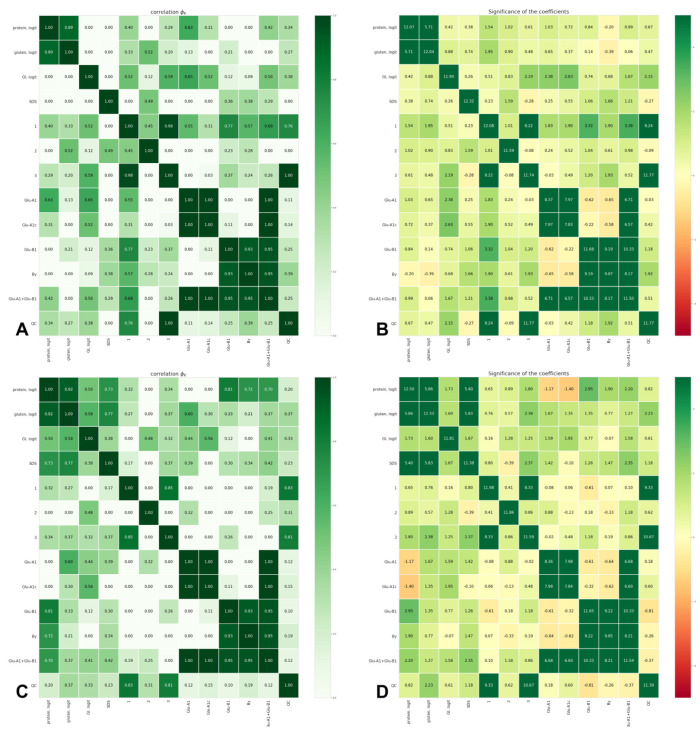
Φk correlation indices (**A**,**C**) and their significance (**B**,**D**) between grain quality of durum wheat grown in 2019–2020 (**A**,**B**) and 2020–2021 (**C**,**D**) and the allelic state of *Glu-A1* and *Glu-B1*: protein, logit—protein content (logit-transformed data); gluten, logit—gluten content (logit-transformed data); GI, logit—gluten index (logit-transformed data); SDS—SDS sedimentation volume; 1—volume increase index; 2—breaking strength; 3—general estimation of pasta; Glu-A1—allelic state of *Glu-A1*; Glu-A1c—presence of *Glu-A1c* or other allele; Glu-B1—allelic state of *Glu-B1*; By—subunit By; Glu-A1 + Glu-B1—allelic combination *Glu-A1* + *Glu-B1*; QC—quality category of pasta.

In both years of observation, the gluten content showed a high correlation with the protein content (Φk 0.89 and 0.92, Figure 3). In 2020–2021, a strong correlation was found between the protein and gluten contents on one side and SDS on the other side (Φk 0.73 and 0.77, respectively, Figure 3). The association between the grain yield and the quality category was absent in both years of the field trials (Φk 0.25 and 0.28). The ranging of accessions, according to the superiority of the quality traits, revealed four accessions from the competitive variety trial nursery (737/11, 21545t, 589/13, 3336h6-13-162) that overcame the standards, Krucha and Krupinka, in quality parameters and, in some cases, in grain yield in one or both years of the field experiments (Table 3).

### 3.3. Gluten Index

The effect of *Glu-A1* on the gluten index was statistically significant only in 2019–2020 (Φk 0.65, Figure 3); the gluten index in the *Glu-A1a* genotypes was significantly higher compared with the *Glu-A1c* genotypes by 26.1 pp (33.6%). In 2020–2021, the *Glu-A1a*-carriers exceeded those with *Glu-A1c* by 10.5 pp (11.2%; the difference is statistically insignificant). In both years, the *Glu-A1c* genotypes were the closest to the HQ category (gluten index values of 51.6 and 83.3, respectively). The effect of *Glu-B1* was statistically significant in 2020–2021; the highest gluten index value was observed in the *Glu-B1al* genotypes, significantly outperforming the *Glu-B1e* genotypes by 31.8 pp (33.4%); By8- and By16- had higher gluten indexes than By20-carriers, although were statistically insignificant. The *Glu-B1b* genotypes were the closest to the HQ category (gluten index values of 53.3 and 82.2). In 2019–2020, the *Glu-A1a* + *Glu-B1f* (89.2%) genotype had the highest gluten index value (89.2%), statistically significantly outperforming the *Glu-A1c* + *Glu-B1e* (47.4%) and *Glu-A1c* + *Glu-B1b* (48.8%) genotypes by 41.8 pp and 40.4 pp (46.9% and 45.3%), respectively. In 2020–2021, the highest gluten index value was demonstrated by the *Glu-A1a* + *Glu-B1f* (99.2%) and *Glu-A1c* + *Glu-B1al* (94.7%) genotypes, exceeding *Glu-A1c* + *Glu-B1e* (53.7%) by 45.5 pp and 41.0 pp (45.9% and 43.3%), respectively. The carriers of *Glu-A1c* + *Glu-B1b* in 2019–2020 and *Glu-A1c* + *Glu-B1f* in 2020–2021 were the closest to the HQ category (Table 2).

### 3.4. Gluten Content

The effect of *Glu-A1* on the gluten content in both years was statistically insignificant. *Glu-B1* significantly affected the gluten content in 2020–2021 only; accessions with By16 (24.7%) showed the highest gluten content, statistically significantly outperforming those with By20 and By8 by 2.5 pp and 2.6 pp (10.1% and 10.5%), respectively. The *Glu-B1f* genotypes (24.7%) were significantly superior to the *Glu-B1b* genotypes by 2.9 pp (11.7%). The carriers of *Glu-A1c* + *Glu-B1al* were the closest to the HQ category in both years, while *Glu-A1a* + *Glu-B1f* in 2020–2021 only (Table 2).

### 3.5. SDS Sedimentation Volume

In 2020–2021, SDS was significantly influenced by the *Glu-B1* allelic state; accessions with By16 subunit had an SDS value statistically significantly higher than those with By20 and By8 subunits by 4.5 and 3.3 units (11.8% and 8.6%), respectively. This effect was reflected in statistically significant differences between the *Glu-A1c* + *Glu-B1f* and *Glu-A1c* + *Glu-B1e* genotypes by 5.3 pp (13.4%, Table 2; Φk 0.41, Figure 3). The carriers of *Glu-A1c* + *Glu-B1d* in 2019–2020 and *Glu-A1c* + *Glu-B1al* in 2020–2021 were the closest to the HQ category (Table 2).

### 3.6. Protein Content

In both years of assessment, the trend of higher protein content in the *Glu-B1f* and *Glu-B1al* genotypes was observed, although the statistical significance of differences was observed only in 2020–2021 (Φk 0.81, Figure 3); *Glu-B1al* and *Glu-B1f* significantly outperformed *Glu-B1b* by 0.7 pp and 0.9 pp (5.2% and 6.6%), respectively. In 2020–2021, the *Glu-A1c* + *Glu-B1f* (13.9%) and *Glu-A1c* + *Glu-B1al* (13.5%) genotypes significantly outperformed the *Glu-A1c* + *Glu-B1b* (12.6%) genotype by 1.3 pp and 0.9 pp (9.3% and 6.7%), respectively (Table 2; Φk 0.70, Figure 3). The By subunit carriers (Φk 0.72, Figure 3) also showed an effect on protein content in 2020–2021; accessions with By16 outperformed those with By8 by 0.7 pp (5.1%). The carriers of *Glu-A1c* + *Glu-B1al* in 2019–2020 and *Glu-A1c* + *Glu-B1e* in 2020–2021 were the closest to the HQ category (Table 2).

### 3.7. Volume Increase Index

The effect of *Glu-B1* on the volume increase index in 2019–2020 was significant; accessions with By16 and By8 subunits significantly outperformed those with By20 by 0.30 units and 0.25 units (7.6% and 6.4%), respectively, and the *Glu-B1f*, *Glu-B1al* and *Glu-B1b* genotypes had statistically significantly higher volume increase index values than *Glu-B1e* by 0.30, 0.28 and 0.24 units, respectively (Table 2, Φk 0.77 for *Glu-B1*, Figure 3). The carriers of *Glu-A1b* + *Glu-B1b* and *Glu-A1c* + *Glu-B1e* in 2019–2020 and *Glu-A1a* + *Glu-B1f* in 2020–2021 were the closest to the HQ category (Table 2).

## 4. Discussion

**Figure 4:**
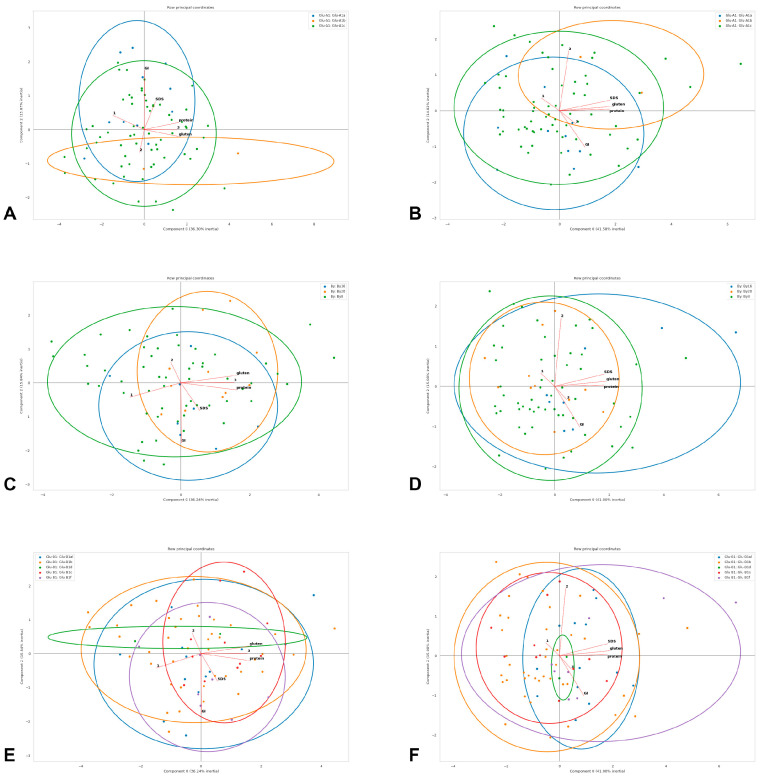
Principal component analysis of quality traits in the studied winter durum wheat accessions with different *Glu-A1* (**A**,**B**), By (**C**,**D**) and *Glu-B1* (**E**,**F**) genotypes estimated in 2019–2020 (**A**,**C**,**E**) and 2020–2021 (**B**,**D**,**F**): protein—protein content (logit-transformed data); gluten—gluten content (logit-transformed data); GI—gluten index (logit-transformed data); SDS—SDS sedimentation volume; 1—volume increase index; 2—breaking strength; 3—general estimation of pasta. Genotypes are shown with dots of different colours deciphered in the upper frame in each diagram.

The analyses performed in the present study revealed the structure of the winter durum wheat collection. The most frequent allele was *Glu-A1c*, which is a common feature in many durum wheat collections [32,40,41]; here, a definite positive effect of the *Glu-A1a* allele and a negative effect of *Glu-A1c* on the gluten index were shown, which is visible by the shape of the corresponding genotype clouds and the gluten index vector in the PCA biplots (Figure 4) and is consistent with other communications [13,14,42]. In the studied collection, accessions that were classified as the HQ category had, on average, a lower gluten index value, to which corresponds *Glu-A1c*, which could explain its prevalence in our collection.

In our collection, *Glu-B1b* showed the highest frequency, followed by *Glu-B1al*, *Glu-B1e* and *Glu-B1f*. In similar studies, the *Glu-B1b*, *Glu-B1d* and *Glu-B1e* alleles had the highest frequencies [32,40]. Thus, the predominance of *Glu-B1al* and *Glu-B1f* is unusual for our collection. This may be explained either by the presence of bread wheat in the pedigrees of the studied durum wheat accessions or by the fact that the grain of winter durum wheat is developed under other conditions than that of spring durum wheat. The *Glu-B1al* allele (Bx7^OE^ + By8) carries a Bx7^OE^ subunit that is overexpressed as a result of Bx7 gene duplication, which is difficult or impossible to detect using SDS-PAGE; its detection requires a separate PCR marker for the boundary region between the second Bx7 copy and its surround [43]. When PCR markers with primers designed for the internal Bx7 region are used, genotypes may be erroneously identified as Bx7 and, thus, as *Glu-B1b*. In our study, to detect *Glu-B1al*, a combination of protein markers, PCR markers for the internal Bx7 region and a KASP marker distinguishing Bx7^OE^ from the other alleles was used. Thus, a reassessment of *Glu-B1al* occurrence in other durum wheat collections using a combination of protein and PCR markers might be necessary.

In most durum wheat studies, the *Glu-B1b* (Bx7 + By8) and *Glu-B1d* (Bx6 + By8) alleles had a positive effect on gluten strength [14]. In our study, *Glu-B1f* (Bx13 + By16) and *Glu-B1al* (Bx7^OE^ + By8) had a positive effect on protein and gluten content (in 2020–2021 statistically significant; in 2019–2020, only the general trend of positive influence is visible from the genotype clouds in the PCA biplot, Figure 4). In [44], the highest gluten strength is observed among the *Glu-B1f* durum wheat genotypes, while, in [45], *Glu-B1al* was found to confer a greater dough-mixing strength and mixograph tolerance in winter durum wheat. At the same time, it is worth noting that the accessions with *Glu-B1d* and *Glu-B1e* demonstrated the highest general pasta estimation in both years, which demonstrated their contribution to a lower volume increase index associated with a better starch–protein interaction during cooking.

Based on the present study, *Glu-A1a*, *Glu-B1f* and *Glu-B1al* can be considered preferable in winter durum breeding programs to increase the gluten index, SDS and the protein and gluten contents, while *Glu-B1d* and *Glu-B1e* can be recommended to optimize the volume increase index and *Glu-A1c* can be recommended to optimize the gluten index. In the bread wheat, the generally high associations with a greater gluten strength, good extensibility and a higher bread loaf volume made from common bread wheat are demonstrated by the genotypes with *Glu-A1a*, *Glu-A1b*, *Glu-B1al*, *Glu-B1i*, *Glu-B1f* and *Glu-D1d*, whereas *Glu-A1c*, *Glu-B1a*, *Glu-B1d* and *Glu-D1a* generally confer an overall poor quality [46]. Therefore, even bread wheat germplasm carrying “non-desirable” for breadmaking quality alleles *Glu-A1c* and *Glu-B1d* can be introduced into winter durum wheat programs, depending on their aims.

Environmental effects such as temperature and precipitation could affect the allelic performance of *Glu-1* genes on grain and pasta quality, especially during the grain filling period [32,47,48]. Indeed, in 2019–2020, the main effects were associated with the influence of *Glu-A1* on the gluten index, while, in 2020–2021, the effects of *Glu-B1* on SDS and the protein and gluten contents were observed. Nevertheless, the main positive trends of *Glu-A1a, Glu-B1f* and *Glu-B1al* on the grain protein traits and *Glu-A1c, Glu-B1d* and *Glu-B1e* on the general pasta estimation remained stable between years.

The accessions with a lower volume index increase demonstrated the highest quality category in both years of estimation (the opposite direction of the corresponding vectors in the PCA biplots, Figure 4). A negative correlation between the volume index increase and the general pasta estimation in winter durum wheat has previously been found in [22]. Thus, it can be assumed that there is a *Glu-A1-* and *Glu-B1*-independent genetic system that consistently affects the volume index increase and the overall pasta quality. In 2019–2020, the quality category and protein and gluten contents appeared to be co-dependent, but, in 2020–2021, these vectors in the PCA biplot demonstrated independence (Figure 4). Such a system could include genes for low-molecular-weight glutenins, starch synthesis and pigments, and requires an additional individual study.

## 5. Conclusions

In the studied collection of winter durum wheat collection estimated for grain and pasta quality by the results of a two-year field experiment under conditions of Krasnodar, the *Glu-A1* and *Glu-B1* loci had different effects on the studied parameters in different years of the assessment; the gluten index was influenced by *Glu-A1* in 2019–2020, while, in 2020–2021, it was influenced by *Glu-B1*. *Glu-B1* had a significant effect on the volume increase index only in 2019–2020 and on the SDS sedimentation volume and gluten and protein contents only in 2020–2021. At the same time, the following general trends in the influence of *Glu-1* genotypes on quality parameters were revealed: *Glu-A1a, Glu-B1al* and *Glu-B1f* had a positive effect on the quality and quantity of protein and gluten, while the *Glu-A1c* + *Glu-B1al* genotypes were closest to the high-quality category in the gluten index, SDS and the protein and gluten contents.

## Figures and Tables

**Table 1:** Allelic state of *Glu-A1* and *Glu-B1* in the studied collection of durum wheat and their values of general pasta estimation (GPE).

Accession	*Glu-A1*	*Glu-B1*	GPE		Accession	*Glu-A1*	*Glu-B1*	GPE	
			2019–2020	2020–2021				2019–2020	2020–2021
Afina	*Glu-A1c*	*Glu-B1b*	4.5	4	MV Pennedur	*Glu-A1c*	*Glu-B1h*	4.5	4.5
Agat Donskoy	*Glu-A1c*	*Glu-B1b*	4	4	MVTD 12-97	*Glu-A1c*	*Glu-B1al*	4.5	4
Akveduk	*Glu-A1c*	*Glu-B1b*	3.5	4	MVTD 12-99	*Glu-A1c*	*Glu-B1e*	4.5	4.5
Alena	*Glu-A1c*	*Glu-B1b*	4.5	4.5	MVTD 15-98	*Glu-A1c*	*Glu-B1e*	4.5	4.5
Altana	*Glu-A1c*	*Glu-B1b*	4	4	Oniks	*Glu-A1a*	*Glu-B1b*	4.5	4
AMA 11855t85-90	*Glu-A1c*	*Glu-B1al*	3.5	3.5	Pandur	*Glu-A1c*	*Glu-B1b*	4	4.5
AMA 15908ds1122-07	*Glu-A1c*	*Glu-B1f*	3.5	3.5	Perlina odesskaya	*Glu-A1c*	*Glu-B1e*	4.5	3.5
Amazonka	*Glu-A1c*	*Glu-B1f*	4	3.5	Pributkova	*Glu-A1c*	*Glu-B1b*	3.5	3.5
Andromeda	*Glu-A1c*	*Glu-B1b*	3.5	4	Prikumchanka	*Glu-A1c*	*Glu-B1b*	4	3.9
Bagryanitsa (Italikum)	*Glu-A1c*	*Glu-B1b*	4.5	3.5	Rodur	*Glu-A1c*	*Glu-B1al*	3.5	4
Belgorodskaya yantarnaya	*Glu-A1c*	*Glu-B1b*	3.5	4	Rumyniya DF-00091-14-002	*Glu-A1c*	*Glu-B1al*	4	3.8
Burshtyn	*Glu-A1c*	*Glu-B1e*	4.5	4	Rumyniya DF-01058-1-101	*Glu-A1c*	*Glu-B1al*	3.5	4
Condur	*Glu-A1c*	*Glu-B1al*	4	3	Shulyndinka	*Glu-A1c*	*Glu-B1b*	3.5	4
DF-104/85	*Glu-A1c*	*Glu-B1b*	4.5	4	Sin’ora	*Glu-A1c*	*Glu-B1f*	4	3.8
Diona	*Glu-A1a*	*Glu-B1b*	4.5	4	Stepnoy yantar’	*Glu-A1c*	*Glu-B1al*	3.5	3.5
Dnepryana	*Glu-A1c*	*Glu-B1b*	4	4	Temnyy yantar’	*Glu-A1c*	*Glu-B1b*	3.5	4.5
Dobryana	*Glu-A1c*	*Glu-B1e*	4	4.5	Teyya	*Glu-A1c*	*Glu-B1e*	4.5	4.5
Donchanka	*Glu-A1a*	*Glu-B1e*	4	4	Uniya	*Glu-A1c*	*Glu-B1e*	4	4
Elidur	*Glu-A1c*	*Glu-B1al*	4	4	Uslada	*Glu-A1c*	*Glu-B1b*	4	3.9
Eyrena	*Glu-A1a*	*Glu-B1b*	4	3.5	Wintergold	*Glu-A1c*	*Glu-B1b*	3.5	4.5
Khar’kovskaya 32	*Glu-A1a*	*Glu-B1b*	3.5	4	Yagut	*Glu-A1c*	*Glu-B1al*	4	4.3
Kiprida	*Glu-A1c*	*Glu-B1b*	4.5	3.5	Yakhont (760/10)	*Glu-A1a*	*Glu-B1f*	4	4
Kontinent	*Glu-A1c*	*Glu-B1b*	4	4.5	Yubilyarka (330/10)	*Glu-A1a*	*Glu-B1b*	4	3.9
Kordon	*Glu-A1c*	*Glu-B1f*	4	4	Zhivitsa	*Glu-A1c*	*Glu-B1e*	4	4
Koshelevskaya	*Glu-A1c*	*Glu-B1b*	3.5	4.5	Zolotko	*Glu-A1c*	*Glu-B1b*	4.5	4
Kristall 2	*Glu-A1b*	*Glu-B1b*	4.5	4	Zoloto Dona	*Glu-A1c*	*Glu-B1b*	4	4.4
Kristella	*Glu-A1a*	*Glu-B1al*	4	4	533/14	*Glu-A1c*	*Glu-B1e*	4	4
Krucha	*Glu-A1c*	*Glu-B1b*	4	4	589/13	*Glu-A1a*	*Glu-B1b*	4	4.5
Krupinka	*Glu-A1c*	*Glu-B1b*	4	4	655/13	*Glu-A1c*	*Glu-B1b*	4	4
Kurant	*Glu-A1a*	*Glu-B1b*	4	3.9	737/11	*Glu-A1a*	*Glu-B1f*	4	4.5
Laska	*Glu-A1c*	*Glu-B1e*	4	3.5	796/11	*Glu-A1c*	*Glu-B1b*	4	4.5
Lazurit	*Glu-A1c*	*Glu-B1b*	4	3.5	21545t	*Glu-A1c*	*Glu-B1f*	4	4
Leukurum 21	*Glu-A1c*	*Glu-B1b*	4.5	4	3336h6-13-162	*Glu-A1c*	*Glu-B1b*	4	4
Leukurum 36	*Glu-A1c*	*Glu-B1b*	4	4	3561h28	*Glu-A1b*	*Glu-B1b*	4	4
Makar	*Glu-A1c*	*Glu-B1b*	3.5	4.5	3591h96	*Glu-A1c*	*Glu-B1b*	4	4.5
Martondur 1	*Glu-A1c*	*Glu-B1al*	4	3.8	3622h46	*Glu-A1c*	*Glu-B1d*	4	4.5
MV Hundur	*Glu-A1c*	*Glu-B1al*	4.5	4.5	3680h62	*Glu-A1c*	*Glu-B1d*	4.5	4
MV Makaroni	*Glu-A1c*	*Glu-B1al*	4.5	4.5	3796h33	*Glu-A1c*	*Glu-B1f*	3.5	3.8

**Table 2:** Mean values of quality traits of 76 winter durum wheat accessions. Mean values designated with the same letters have no significant differences within each group, as calculated using Tukey’s criterion. For quality category (QC, HQ—high quality, MQ—medium quality, LQ—low quality), the number of accessions is shown for the 2019–2020/2020–2021 years of the field trial.

Locus/ Protein	Allele/ Subunit	Number of Accessions	Volume Increase Index		Breaking Strength		General Pasta Estimation		Gluten Index, %		SDS		Gluten Content, %		Protein Content, %	
			2019–2020	2020–2021	2019–2020	2020–2021	2019–2020	2020–2021	2019–2020	2020–2021	2019–2020	2020–2021	2019–2020	2020–2021	2019–2020	2020–2021
*Glu-A1*	*Glu-A1a*	11	3.88	4.02	804	788	4.05	4.03	77.7 ᵃ	93.8	39.7	34.4	22.4	22.4	13.6	12.9
	*Glu-A1b*	2	3.55	3.95	865	871	4.25	4.00	47.2 ^a^ᵇ	93.8	41.1	39.8	25.0	24.9	14.6	13.5
	*Glu-A1c*	63	3.89	3.97	818	820	4.02	4.03	51.6 ᵇ	83.3	42.0	35.1	23.2	22.4	13.7	13.0
*Glu-A1c*	*Glu-A1a + Glu-A1b*	13	3.83	4.01	813	801	4.08	4.02	73.9 ^a^	93.8	39.9 ᵇ	35.3	22.8	22.7	13.7	13.0
	*Glu-A1c*	63	3.89	3.97	818	820	4.02	4.03	51.6 ^b^	83.3	42.0 ᵃ	35.1	23.2	22.4	13.7	13.0
By	By16	8	3.97 ᵃ	4.04	840	812	3.88	3.89	67.9	92.4	43.4	38.2 ᵃ	23.7	24.7 ᵃ	14.1	13.6 ᵃ
	By20	11	3.67 ᵇ	3.95	802	813	4.23	4.09	47.5	63.5	42.8	33.7 ᵇ	23.2	22.2 ᵇ	13.6	13.0 ᵃᵇ
	By8	56	3.92 ᵃ	3.98	816	817	4.00	4.03	55.5	87.3	41.2	34.9 ᵇ	23.0	22.1 ᵇ	13.6	12.9 ᵇ
*Glu-B1*	*Glu-B1al*	13	3.95 ^a^	4.02	818	824	3.96	3.92	63.5	95.3 ᵃ	41.1	36.1	23.6	23.2 ᵃᵇ	13.9	13.4 ᵃ
	*Glu-B1b*	41	3.91 ^a^	3.97	815	814	4.00	4.06	53.3	82.2 ^a^ᵇ	41.3	34.6	22.9	21.8 ᵇ	13.5	12.7 ᵇ
	*Glu-B1d*	2	3.85 ᵃᵇ	3.90	825	832	4.25	4.25	45.4	95.8 ᵃᵇ	41.3	33.8	20.8	22.5 ᵃᵇ	13.1	12.9 ᵃᵇ
	*Glu-B1e*	11	3.67 ^b^	3.95	802	813	4.23	4.09	47.5	63.5 ᵇ	42.8	33.7	23.2	22.2 ^a^ᵇ	13.6	13.0 ᵃᵇ
	*Glu-B1f*	8	3.97 ^a^	4.04	840	812	3.88	3.89	67.9	92.4 ᵃᵇ	43.4	38.2	23.7	24.7 ᵃ	14.1	13.6 ᵃ
*Glu-A1 + Glu-B1*	*Glu-A1a Glu-B1b*	7	3.89 ᵃᵇ	4.07	819	783	4.07	3.97	73.7 ᵃᵇ	82.8 ᵃᵇ	39.2	34.4 ᵃᵇ	22.2	22.0 ᵃᵇ	13.4	12.7 ^ab^
	*Glu-A1a Glu-B1f*	2	3.85 ᵃᵇ	3.80	782	804	4.00	4.25	89.2 ᵃ	99.2 ^a^	39.4	34.0 ᵃᵇ	23.6	23.2 ᵃᵇ	14.1	12.9 ᵃᵇ
	*Glu-A1b Glu-B1b*	2	3.55 ᵃᵇ	3.95	865	871	4.25	4.00	47.2 ^a^ᵇᶜ	93.8 ᵃᵇ	41.1	39.8 ᵃᵇ	25.0	24.9 ᵃᵇ	14.6	13.5 ᵃᵇ
	*Glu-A1c Glu-B1al*	12	3.96 ^a^	4.02	822	830	3.96	3.91	60.0 ^a^ᵇᶜ	94.7 ^a^	41.1	36.1 ᵃᵇ	23.8	23.2 ᵃᵇ	13.9	13.5 ^a^
	*Glu-A1c Glu-B1b*	32	3.94 ^a^	3.94	810	817	3.97	4.08	48.8 ᶜ	80.9 ᵃᵇ	41.7	34.3 ᵇ	22.9	21.5 ᵇ	13.5	12.6 ^b^
	*Glu-A1c Glu-B1d*	2	3.85 ᵃᵇ	3.90	825	832	4.25	4.25	45.4 ^a^ᵇᶜ	95.8 ᵃᵇ	41.3	33.8 ᵃᵇ	20.8	22.5 ᵃᵇ	13.1	12.9 ᵃᵇ
	*Glu-A1c Glu-B1e*	10	3.65 ^b^	3.93	806	812	4.25	4.10	47.4 ^b^ᶜ	53.7 ^b^	42.8	33.6 ᵇ	23.3	22.2 ^a^ᵇ	13.6	13.0 ᵃᵇ
	*Glu-A1c Glu-B1f*	6	4.02 ^a^	4.12	860	815	3.83	3.77	57.3 ᵃᵇ^c^	84.6 ᵃᵇ	44.8	39.6 ᵃ	23.7	25.2 ^a^	14.1	13.9 ^a^
QC	3.0–3.5 (LQ)	16/12	4.12 ^a^	4.30 ^a^	843	787	3.50 ᶜ	3.46 ᶜ	42.6 ᵇ	80.9	40.5	33.9	22.4 ᵇ	22.1	13.3 ᵇ	12.9
	3.8–4.1 (MQ)	40/43	3.93 ᵇ	4.02 ᵇ	812	824	4.00 ᵇ	3.97 ᵇ	64.0 ᵃ	87.1	42.3	35.1	22.9 ᵇ	22.3	13.7 ᵃ	13.0
	4.3–4.5 (HQ)	20/21	3.60 ^c^	3.70 ^c^	809	821	4.50 ᵃ	4.49 ᵃ	49.0 ᵇ	85.6	41.4	35.9	24.1 ᵃ	22.9	13.9 ᵃ	13.1

**Table 3:** The accessions with the superior quality parameters compared with the standards, Krucha and Krupinka, as revealed by two-year field trials.

Accession	2019–2020				2020–2021			
	Gluten Index, %	Gluten Content, %	General Pasta Estimation	Grain Yield, kg per 4.5 m^2^ Plot	Gluten Index, %	Gluten Content, %	General Pasta Estimation	Grain Yield, kg per 4.5 m^2^ Plot
Krucha	56.4	22.1	4.0	4.1	79.1	22.9	4.0	3.2
Krupinka	69.2	21.7	4.0	3.9	87.8	22.9	4.0	3.3
737/11	90.2	25.2	4.0	4.6	99.4	22.9	4.5	3.5
21545t	80.0	26.6	4.0	3.1	92.3	30.5	4.0	2.4
589/13	75.7	25.5	4.0	4.6	99.7	25.4	4.5	2.9
3336h6-13-162	53.7	26.6	4.0	3.7	99.0	27.8	4.0	3.5

## Data Availability

Data are contained within the article and Supplementary Materials.

## Supplementary Materials

The following supporting information can be downloaded at: https://www.mdpi.com/article/10.3390/foods12071436/s1, Supplementary Table S1: Origin and release year of winter durum wheat accessions.
